# Combating Carbapenem-Resistant *Enterobacterales*: Comparative *In Vitro* Efficacy of Aztreonam–Avibactam and Ceftazidime–Avibactam and Distribution of Carbapenemase Genes

**DOI:** 10.3390/antibiotics15080724

**Published:** 2026-07-25

**Authors:** Melike Yaşar Duman, Mervenur Kanat, Elif Seren Tanrıverdi, Feriha Çilli, Şöhret Aydemir

**Affiliations:** 1 Department of Medical Microbiology, Faculty of Medicine, Ege University, 35100 İzmir, Türkiye; mervenur.kanat@ege.edu.tr (M.K.); feriha.cilli@ege.edu.tr (F.Ç.); sohret.aydemir@ege.edu.tr (Ş.A.); 2 Department of Medical Microbiology, Faculty of Medicine, İnönü University, 44280 Malatya, Türkiye; seren.tanriverdi@inonu.edu.tr

**Keywords:** carbapenem-resistant *Enterobacterales*, aztreonam–avibactam, ceftazidime–avibactam, *bla*_NDM_, *bla*_OXA-48_, carbapenemase, synergy testing, whole-genome sequencing

## Abstract

**Background:** Carbapenem-resistant *Enterobacterales* (CRE) are a major therapeutic challenge, particularly in settings where metallo-β-lactamases are prevalent. Aztreonam–avibactam (AZA) may provide activity against metallo-β-lactamase-producing isolates, whereas ceftazidime–avibactam (CZA) has limited activity against the isolates that harbor these enzymes. This study aimed to evaluate the in vitro activity of aztreonam–avibactam and ceftazidime–avibactam against invasive carbapenem-resistant *Enterobacterales* isolates and to characterize the distribution of carbapenemase genes in a tertiary-care center in Türkiye. **Methods:** A total of 100 non-duplicate CRE isolates recovered from blood cultures and sterile body fluids between January 2024 and January 2026 were included. Species identification and routine antimicrobial susceptibility testing were performed using MALDI-TOF MS and an automated system. CZA and AZA MICs were determined by gradient diffusion testing, whereas CZA–aztreonam synergy was assessed separately using a disk/gradient diffusion-based method. Carbapenemase genes were detected by real-time PCR. Whole-genome sequencing was performed for the two AZA-resistant *Escherichia coli* isolates. **Results:** The isolate collection was dominated by *Klebsiella pneumoniae* (87.0%), and most isolates were recovered from blood cultures (80.0%). CZA susceptibility was observed in 31.0% of isolates, with MIC_50/90_ values of 256/256 mg/L. In contrast, AZA showed high in vitro activity, with 98.0% of isolates categorized as susceptible and MIC_50/90_ values of 0.25/0.50 mg/L. CZA–aztreonam synergy was detected in 98.0% of isolates. Qualitative CZA–aztreonam synergy testing did not fully concord with direct AZA MIC-based susceptibility categorization: one AZA-resistant isolate showed a positive synergy result, whereas one synergy-negative isolate remained AZA-susceptible. Carbapenemase genes were detected in 99.0% of isolates; *bla*_NDM_ was the most frequent gene (81.0%), followed by *bla*_OXA-48_ (72.0%), *bla*_KPC_ (14.0%), and *bla*_VIM_ (4.0%). The most common carbapenemase profile was *bla*_NDM_ + *bla*_OXA-48_ (62.0%). *bla*_NDM_ carriage was strongly associated with CZA resistance, and CZA MICs were significantly higher among *bla*_NDM_-positive isolates. The two AZA-resistant *E. coli* isolates belonged to ST500 and ST410, and both carried a YRIK insertion in PBP3; one of them additionally showed *ompF* disruption associated with a 4 bp insertion. **Conclusions:** AZA demonstrated potent in vitro activity against invasive CRE isolates in this setting, whereas CZA activity was substantially limited, likely reflecting the high prevalence of *bla*_NDM_ and frequent carbapenemase co-carriage. These findings support the potential value of AZA in regions with emerging metallo-β-lactamase predominance and highlight the importance of local molecular surveillance to guide antimicrobial strategies.

## 1. Introduction

The rise of antimicrobial resistance (AMR) has been recognized by the World Health Organization (WHO) as one of the most serious threats to global public health. Carbapenem-resistant *Enterobacterales* (CRE) were initially classified within the “critical priority” tier of pathogens requiring the urgent development of new antimicrobial agents by WHO in 2017, and they continue to retain this highest-urgency designation in the updated 2024 WHO Bacterial Priority Pathogens List [1,2]. CRE are defined as *Enterobacterales* resistant to one or several carbapenem antibiotics [3].

Carbapenem resistance in *Enterobacterales* may arise through several mechanisms, including alterations in penicillin-binding proteins, reduced outer membrane permeability, efflux pump overexpression, and most importantly, the production of carbapenemase enzymes. Carbapenemases are β-lactamases capable of hydrolyzing a broad range of β-lactam antibiotics, including penicillins, cephalosporins, monobactams, and carbapenems. The most frequently encountered carbapenemases in CRE isolates are class A enzymes (such as KPC), class B metallo-β-lactamases (such as NDM, VIM, and IMP), and class D enzymes (such as OXA-48) [4].

The global dissemination of metallo-β-lactamase (MBL)-producing Gram-negative bacteria represents a major therapeutic challenge, as these enzymes hydrolyze most β-lactam antibiotics, including carbapenems, while monobactams such as aztreonam remain relatively stable against MBL-mediated hydrolysis [4]. However, ceftazidime–avibactam, imipenem–relebactam, and meropenem–vaborbactam generally lack in vitro activity against MBL-producing Gram-negative bacilli, as the corresponding β-lactamase inhibitors do not inhibit class B metallo-β-lactamases [5]. This limitation creates a critical gap in current antimicrobial treatment options and highlights the urgent need for new therapeutic strategies.

In contrast, the combination of aztreonam and avibactam has emerged as a promising therapeutic option for infections caused by MBL-producing *Enterobacterales*. Aztreonam remains stable against hydrolysis by MBLs, while avibactam inhibits co-produced serine β-lactamases that would otherwise inactivate aztreonam [6]. Preliminary in vitro studies from different regions have demonstrated potent activity of aztreonam–avibactam against MBL-producing *Enterobacterales*; however, regional data remain limited.

The epidemiology of carbapenemase-producing *Enterobacterales* in Türkiye has been characterized by an OXA-48-endemic background, while recent reports have also documented NDM-producing isolates and multiple-carbapenemase profiles, including OXA-48/NDM and KPC/NDM/OXA-48 co-carriage [7,8,9,10]. In addition to OXA-48-like enzymes, NDM-type metallo-β-lactamases have increasingly been reported in Türkiye, either alone or as part of multiple-carbapenemase profiles, including OXA-48/NDM co-production, which has been associated with hospital outbreaks and poses additional challenges for treatment and infection control [9,10]. These findings indicate a dynamic and evolving epidemiological landscape of carbapenem resistance in Türkiye and highlight the need for continuous surveillance and evaluation of novel antimicrobial combinations active against these resistance mechanisms.

However, available data including isolates from Türkiye remain limited, particularly for invasive carbapenem-resistant *Enterobacterales* collections with local carbapenemase characterization [11]. Since aztreonam–avibactam is not yet available for clinical use in Türkiye, evaluating its potential activity against local CRE isolates is of particular importance.

Therefore, this study aimed to evaluate the in vitro activity of aztreonam–avibactam and ceftazidime–avibactam against invasive carbapenem-resistant *Enterobacterales* isolates and to characterize the distribution of carbapenemase genes in a tertiary-care center in Türkiye.

## 2. Materials and Methods

### 2.1. Study Design and Isolate Selection

This retrospective, observational, cross-sectional in vitro study was conducted at Ege University Faculty of Medicine, Department of Medical Microbiology Laboratory, a tertiary-care center in İzmir, Türkiye. Between 1 January 2024 and 20 January 2026, all CRE strains isolated from blood cultures and sterile body fluids (cerebrospinal fluid, pleural fluid, and peritoneal aspirates) were screened from the institutional microbiology archive. In cases where multiple isolates were obtained from the same patient, only the first isolate was included to avoid duplication. All archived isolates had been stored at −80 °C and were retrieved for the present study. Each isolate was subcultured on 5% sheep blood agar (BioMérieux, France). Isolates that failed to regrow after subculture were excluded from further analysis.

Initially, 101 non-duplicate CRE isolates met the inclusion criteria. During molecular analysis, one isolate repeatedly failed to yield PCR amplification despite three independent DNA extraction and amplification attempts. This isolate was therefore excluded from the study to maintain a consistent denominator across phenotypic susceptibility analyses, molecular analyses, and genotype–phenotype comparisons. Consequently, the final study population consisted of 100 non-duplicate CRE isolates, and all reported percentages were calculated using this final study population.

### 2.2. Bacterial Identification and Carbapenem Resistance Confirmation

In routine laboratory practice, bacterial isolates were identified to the species level using matrix-assisted laser desorption/ionization time-of-flight mass spectrometry (VITEK MS; BioMérieux, Marcy-l’Étoile, France) and antimicrobial susceptibility testing was performed using the VITEK 2 automated system (AST-N420 cards; BioMérieux, Marcy-l’Étoile, France).

When resistance to any carbapenem was detected, gradient diffusion testing (E test, BioMérieux, Marcy-l’Étoile, France) was performed to determine minimum inhibitory concentration (MIC) of the corresponding carbapenem, namely imipenem, meropenem or ertapenem. Results were interpreted according to EUCAST 2025 clinical breakpoints [12].

### 2.3. Aztreonam–Avibactam, Ceftazidime–Avibactam MIC Determination

Isolates were purified by subculturing a single colony onto blood agar plates and incubated at 35 ± 2 °C for 24 h. Bacterial suspensions equivalent to a 0.5 McFarland standard were prepared in sterile saline and inoculated onto Mueller–Hinton agar (BioMérieux, France).

Ceftazidime–avibactam (BioMérieux, France) and aztreonam–avibactam (Liofilchem S.r.l., Roseto degli Abruzzi, Italy) gradient test strips were applied to the agar surface. Plates were incubated under atmospheric conditions for 18–24 h at 35 ± 2 °C. Results were interpreted according to EUCAST 2025 guidelines [12]. For aztreonam–avibactam, *Enterobacterales* isolates were categorized as susceptible at MIC values ≤ 4 mg/L and resistant at MIC values > 4 mg/L.

### 2.4. Ceftazidime–Avibactam/Aztreonam Synergy Testing

**Figure 1:**
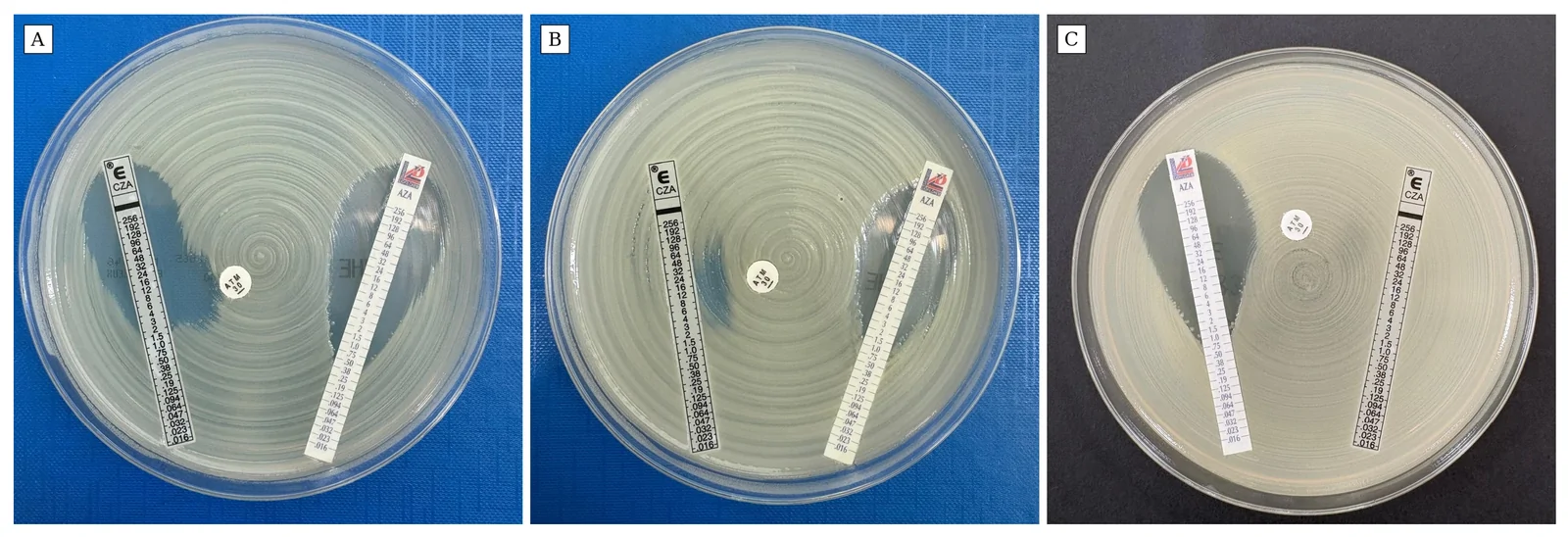
Representative ceftazidime–avibactam/aztreonam synergy test images. Panels (**A**,**B**) demonstrate positive synergy, indicated by enhancement of the aztreonam inhibition zone toward the ceftazidime–avibactam gradient strip, whereas panel (**C**) shows no visible synergy.

Ceftazidime–avibactam/aztreonam synergy was assessed using a disk/gradient diffusion-based method adapted from Taha et al. [13]. A 30-µg aztreonam disk (Oxoid, Thermo Fisher Scientific, Basingstoke, UK) was placed at a 15 mm distance from the center of the disk to the longitudinal midline of the ceftazidime–avibactam gradient strip on the inoculated Mueller–Hinton agar plate [13]. Plates were incubated under atmospheric conditions for 18–24 h at 35 ± 2 °C. Synergy was interpreted visually by a microbiologist according to predefined criteria, based on enhancement of the aztreonam inhibition zone toward the ceftazidime–avibactam strip or the presence of an inverse-D-shaped inhibition pattern (Figure 1).

This assay was used to assess phenotypic interaction between aztreonam and avibactam and was not considered a substitute for direct aztreonam–avibactam MIC determination.

### 2.5. Quality Control and Reference Strains

The following well-characterized reference strains were included as quality controls for antimicrobial susceptibility testing and real-time PCR assays: *Klebsiella pneumoniae* CCUG 56233 (KPC-2), *K. pneumoniae* NCTC 13442 (OXA-48), *K. pneumoniae* NCTC 13443 (NDM-1), *K. pneumoniae* NCTC 13440 (VIM), and *Escherichia coli* NCTC 13476 (IMP).

### 2.6. Real-Time PCR for Carbapenemase Gene Detection

To investigate the molecular mechanisms underlying carbapenem resistance, carbapenemase-encoding genes were analyzed using a polymerase chain reaction (PCR)-based approach. Genomic DNA required for molecular assays was obtained from bacterial culture isolates using a standardized nucleic acid extraction protocol.

### 2.7. DNA Extraction

Genomic DNA was extracted from bacterial isolates using the Bio-Speedy^®^ Fast Nucleic Acid Extraction Kit (Bioeksen R&D Technologies, İstanbul, Türkiye) according to the manufacturer’s instructions.

Fresh bacterial colonies obtained from overnight cultures on blood agar plates were used for nucleic acid extraction. Briefly, 1–3 well-isolated colonies were transferred into sterile microcentrifuge tubes containing Bio-Speedy^®^ lysis buffer (STL-B) and vortexed thoroughly.

Proteinase K was added to facilitate enzymatic cell lysis and protein degradation. The resulting lysates were subsequently processed using a magnetic bead–based automated nucleic acid extraction system (Zybio EXM3000; Zybio Inc., Chongqing, China), enabling selective binding and purification of nucleic acids.

### 2.8. Real-Time PCR Assay

Carbapenemase-encoding genes were detected using the Bio-Speedy^®^ Carbapenem Resistance qPCR Kit (Bioeksen R&D Technologies, Türkiye) according to the manufacturer’s instructions. The assay enables qualitative detection of major carbapenemase genes, including *bla*_KPC_, *bla*_NDM_, *bla*_VIM_, *bla*_IMP_, and *bla*_OXA-48_, as well as *bla*_CTX-M_–type β-lactamases.

Real-time PCR amplification was performed using the Magnetic Induction Cycler (Mic IVD; Bio Molecular Systems, Upper Coomera, Australia) under the thermal cycling conditions recommended by the manufacturer. Fluorescence signals were monitored in the FAM, HEX, ROX, and CY5 channels, corresponding to the respective carbapenemase targets.

Negative and positive controls supplied with the kit were included in each run. In addition, well-characterized ATCC/NCTC reference strains harboring known carbapenemase genes were used as external quality controls to verify assay performance. Each run was considered valid only when both internal and external controls produced the expected results.

Samples showing sigmoidal amplification curves exceeding the predefined threshold values were interpreted as positive for the corresponding carbapenemase gene.

### 2.9. Whole-Genome Sequencing and Bioinformatic Analysis

Whole-genome sequencing (WGS) was performed on two *Escherichia coli* isolates in which resistance to aztreonam–avibactam was detected at the Medical Microbiology Laboratory, Faculty of Medicine, İnönü University. Genomic DNA was extracted using standard protocols suitable for Gram-negative bacteria. Sequencing libraries were prepared using the NEBNext^®^ DNA Library Prep Kit (New England Biolabs, Ipswich, MA, USA) according to the manufacturer’s instructions. WGS was performed on the Illumina NovaSeq 6000 platform (Illumina, San Diego, CA, USA), generating 150 bp paired-end reads. Raw sequencing reads were obtained in FASTQ format, and the mean sequencing depth was approximately 225×.

Initial quality control, genome assembly, annotation, antimicrobial resistance gene detection, and multilocus sequence typing (MLST) were performed using the Bactopia pipeline (v3.2.0) with default parameters (https://bactopia.github.io/). Within Bactopia, read quality control was performed with fastp (v0.23.4) and FastQC (v0.12.1); de novo assembly was performed with Shovill (v1.1.0) using the SKESA (v2.5.1) assembler; genome annotation was performed with Prokka (v1.14.6); antimicrobial resistance and stress-response gene screening was performed with NCBI AMRFinderPlus (v4.0.19, database v4.0.19); and MLST was performed using mlst (v2.23.0; T. Seemann) against the PubMLST database. Genome sketches for taxonomic/contamination checks were generated with Mash (v2.3) and Sourmash (v4.8.2).

Preliminary gene-level comparisons were conducted by extracting relevant coding sequences (*ftsI*, *ompF*, *ompC*) from assembled genomes and performing multiple sequence alignment using Clustal Omega (v1.2.4) via the EMBL-EBI web server (https://www.ebi.ac.uk/jdispatcher/msa/clustalo/ (accessed on 22 July 2026)). Observed sequence differences were subsequently confirmed through reference-based mapping and variant analysis.

For variant-level investigation, raw reads were aligned to the *Escherichia coli* K-12 MG1655 reference genome (RefSeq accession: NC_000913.3) using BWA-MEM (BWA v0.7.17-r1188). Alignment files were sorted and indexed using SAMtools (v1.13). Variant calling and normalization were performed using BCFtools (v1.13).

The ES_73 *ompF* insertion was manually inspected and confirmed by visualization of the corresponding read alignments in Integrative Genomics Viewer (v2.19.7).

Coding sequence (CDS)-level analyses focused on genes previously implicated in reduced aztreonam susceptibility through alterations in target affinity, outer membrane permeability, efflux regulation, or chromosomal β-lactamase expression, including *ftsI* (PBP3), *ompF*, *ompC*, *marR*, *acrR*, *soxR/soxS*, *ompR*, *envZ*, *acrA*, *acrB*, *tolC*, and the *ampC* promoter/attenuator region. Gene coordinates were extracted from the reference genome annotation, and gene-specific variants were retrieved from normalized VCF files. Gene-specific variant extraction, CDS reconstruction, and translation analyses were performed using Python scripts in a Google Colab environment with Python v3.12.13 [GCC 11.4.0].

Mutant CDS sequences were reconstructed in silico based on identified variants and translated to evaluate amino acid substitutions, frameshift mutations, and premature stop codons. Disruptive mutations were defined as frameshift insertions/deletions or premature stop codons predicted to alter the open reading frame and impair protein function. Pairwise nucleotide and amino acid sequence alignments were additionally performed using EMBOSS Stretcher (EMBOSS v6.6.0.0) via the EMBL-EBI platform to support comparative analyses.

### 2.10. Statistical Analysis

Statistical analyses were performed using jamovi version 2.6.44 (The jamovi Project, Sydney, Australia). Categorical variables were summarised as frequencies and percentages and group comparisons were made using Pearson’s chi-square test; Fisher’s exact test was used when expected cell counts were <5. MIC values were non-normally distributed and were therefore summarized as medians and ranges. MIC values between two groups were compared using the Mann–Whitney U test. To account for the frequent co-carriage of carbapenemase genes, multivariable binary logistic regression analysis was performed using CZA resistance as the dependent variable and *bla*_NDM_, *bla*_OXA-48_, *bla*_KPC_, and *bla*_VIM_ carriage as predictor variables. CZA-susceptible isolates were used as the outcome reference category, and gene-negative isolates were used as the reference category for each carbapenemase gene. *bla*_IMP_ was not included in the model because it was not detected in any isolate. Logistic regression results were reported as adjusted odds ratios (aORs) with 95% confidence intervals (CIs). No formal adjustment for multiple comparisons was applied, as the statistical analyses were limited to hypothesis-driven comparisons directly related to the study objectives. A two-sided *p*-value < 0.05 was considered statistically significant.

## 3. Results

### 3.1. Isolate Characteristics

A total of 100 non-duplicate clinical *Enterobacterales* isolates were included in the study. The majority were *Klebsiella pneumoniae* (*n* = 87), followed by *Escherichia coli* (*n* = 6) and members of the *Enterobacter cloacae* complex (*n* = 5; including three isolates reported at the complex level and two identified as *Enterobacter hormaechei*). Single isolates of *Klebsiella aerogenes* (formerly *Enterobacter aerogenes*) and *Citrobacter braakii* (*n* = 1 each) were also identified.

All isolates were recovered from invasive clinical specimens. Most originated from blood cultures (*n* = 80, 80.0%), while the remaining isolates were obtained from sterile body fluids, including peritoneal fluid (*n* = 12, 12.0%), pleural fluid (*n* = 5, 5.0%), and cerebrospinal fluid (*n* = 3, 3.0%).

### 3.2. Antimicrobial Susceptibility and Synergy Findings

Ceftazidime–avibactam susceptibility testing demonstrated that 31 isolates (31.0%) were categorized as susceptible, whereas 69 isolates (69.0%) were resistant according to EUCAST criteria. CZA MIC values ranged from 0.5 to 256 mg/L, with MIC_50_ and MIC_90_ both at 256 mg/L.

Aztreonam–avibactam exhibited markedly high in vitro activity. A total of 98 isolates (98.0%) were categorized as susceptible, while 2 isolates (2.0%) were resistant. AZA MIC values ranged from 0.032 to 32 mg/L, with an MIC_50_ of 0.25 mg/L and an MIC_90_ of 0.50 mg/L.

Synergy testing based on zone enhancement demonstrated positive synergy in 98 isolates (98.0%), whereas 2 isolates (2.0%) showed no evidence of synergy. Among the two isolates with a negative CZA–aztreonam synergy result, one isolate had an AZA MIC of 1 mg/L and remained susceptible according to EUCAST 2025 criteria, whereas the other isolate, ES_73, had an AZA MIC of 32 mg/L and was categorized as AZA-resistant. Thus, absence of visible synergy did not uniformly correspond to AZA resistance. Conversely, the two AZA-resistant isolates had AZA MICs of 8 mg/L and 32 mg/L, respectively; ES_64 showed a positive synergy result, whereas ES_73 was synergy-negative. The synergy-negative isolate with an AZA MIC of 1 mg/L harbored *bla*_NDM_, *bla*_KPC_, and *bla*_CTX-M_, whereas ES_73 carried *bla*_KPC_.

### 3.3. Year-Specific Susceptibility Patterns

Because the 2026 subset included only five isolates and represented a partial-year dataset, these isolates were included in the overall analysis but excluded from year-to-year trend interpretation. Therefore, year-specific comparisons were restricted to 2024 and 2025. In 2024, 20 of 41 isolates (48.8%) were resistant to ceftazidime–avibactam, whereas in 2025, 46 of 54 isolates (85.2%) were resistant. The CZA MIC_50_ and MIC_90_ were 4 mg/L and 256 mg/L in 2024, respectively, and both were 256 mg/L in 2025. For aztreonam–avibactam, in vitro activity remained high in both years, with AZA MIC_50_ and MIC_90_ values of 0.25 mg/L and 0.50 mg/L in both 2024 and 2025.

### 3.4. Distribution of Carbapenemase Genes

Carbapenemase genes were detected in 99 of 100 isolates (99.0%). The most frequently detected gene was *bla*_NDM_ (81 isolates, 81.0%), followed by *bla*_OXA-48_ (72 isolates, 72.0%). *bla*_KPC_ was identified in 14 isolates (14.0%) and *bla*_VIM_ in 4 isolates (4.0%), whereas *bla*_IMP_ was not detected.

**Figure 2:**
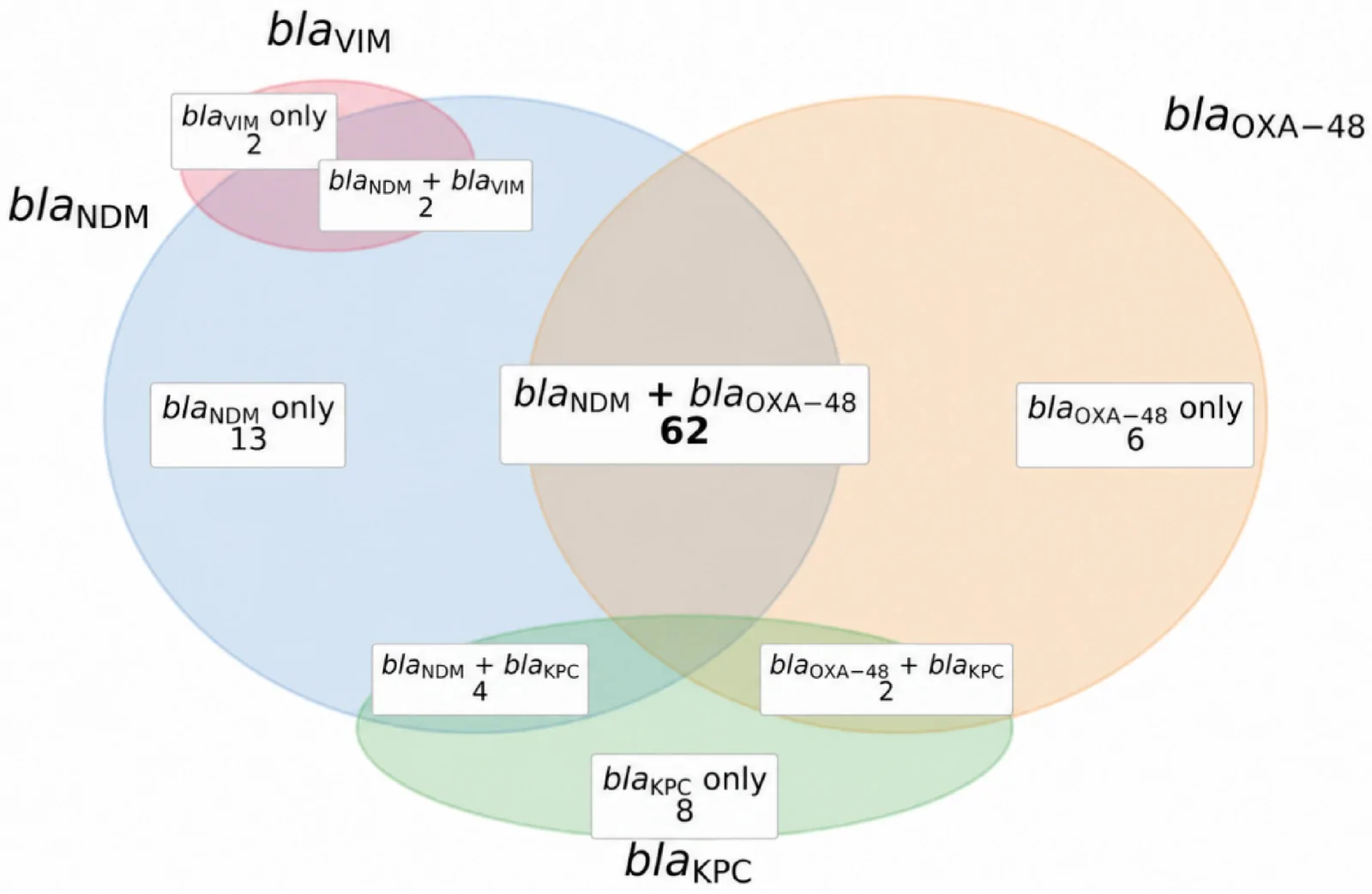
Distribution and co-carriage of carbapenemase genes among carbapenem-resistant *Enterobacterales* isolates. Colored circles represent carbapenemase gene categories: blue, *bla*_NDM_; orange, *bla*_OXA-48_; green, *bla*_KPC_; and pink, *bla*_VIM_.

Among the isolates, the most common carbapenemase gene combination was *bla*_NDM_ + *bla*_OXA-48_, detected in 62 isolates (62.0%). Co-production of multiple carbapenemases was frequently observed, whereas single carbapenemase gene carriage occurred less commonly. The distribution and co-carriage of carbapenemase genes among carbapenem-resistant *Enterobacterales* isolates are presented in Figure 2. The ESBL gene CTX-M was detected in 80 isolates (80.0%). One isolate carried *bla*_CTX-M_ alone without detection of any carbapenemase gene.

The mutually exclusive carbapenemase gene profiles were as follows: *bla*_NDM_ + *bla*_OXA-48_, 62 isolates (62.0%); *bla*_NDM_ alone, 13 isolates (13.0%); *bla*_KPC_ alone, 8 isolates (8.0%); *bla*_OXA-48_ alone, 6 isolates (6.0%); *bla*_NDM_ + *bla*_KPC_, 4 isolates (4.0%); *bla*_VIM_ alone, 2 isolates (2.0%); *bla*_OXA-48_ + *bla*_KPC_, 2 isolates (2.0%); and *bla*_NDM_ + *bla*_OXA-48_ + *bla*_VIM_, 2 isolates (2.0%). One isolate carried *bla*_CTX-M_ alone without detection of any carbapenemase gene. No *bla*_IMP_-positive isolate was detected.

### 3.5. Genotype–Phenotype Associations

Of the 69 CZA-resistant isolates, 67 (97.1%) harbored *bla*_NDM_ and 54 (78.3%) *bla*_OXA-48_, whereas *bla*_KPC_ and *bla*_VIM_ were each detected in 3 isolates (4.3%). Also, 60 (87.0%) isolates had *bla*_CTX-M_. Co-production of carbapenemase genes was common among CZA-resistant isolates, most frequently *bla*_NDM_ + *bla*_OXA-48_ (*n* = 52, 75.4%), followed by *bla*_NDM_ + *bla*_KPC_ (*n* = 2, 2.9%) and the triple combination *bla*_NDM_ + *bla*_OXA-48_ + *bla*_VIM_ (*n* = 2, 2.9%).

Susceptibility rates varied according to carbapenemase gene distribution (Table 1). CZA resistance was strongly associated with *bla*_NDM_ carriage, whereas aztreonam–avibactam retained activity against the majority of the isolates regardless of carbapenemase profile.

Among CZA-susceptible isolates (*n* = 31), *bla*_NDM_ was detected in 14 (45.2%), *bla*_OXA-48_ in 18 (58.1%), and *bla*_CTX-M_ in 20 (64.5%) isolates. In contrast, *bla*_KPC_ and *bla*_VIM_ were identified in 11 (35.5%) and 1 isolate (3.2%), respectively. Gene co-production patterns in this group included *bla*_NDM_ + *bla*_OXA-48_ (*n* = 10, 32.3%), *bla*_NDM_ + *bla*_KPC_ (*n* = 2, 6.5%), and *bla*_KPC_ + *bla*_OXA-48_ (*n* = 2, 6.5%). No isolate in either group carried *bla*_IMP_.

The presence of *bla*_NDM_ was strongly associated with CZA resistance (χ^2^ = 37.5, *p* < 0.001). Conversely, *bla*_KPC_ was more frequently detected among CZA-susceptible isolates (χ^2^ = 17.2, *p* < 0.001).

**Figure 3:**
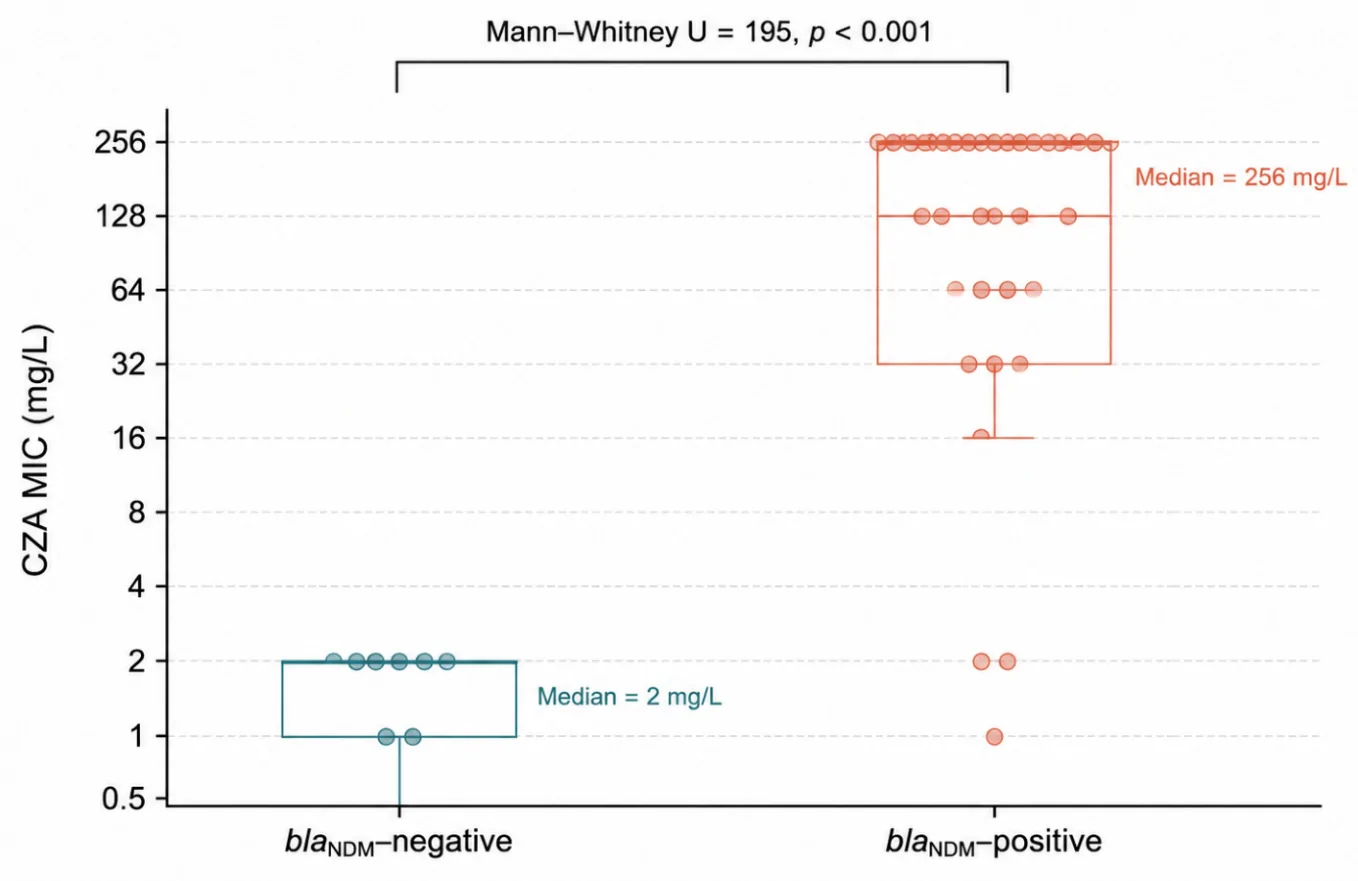
Distribution of ceftazidime–avibactam MIC values according to *bla*_NDM_ carriage among carbapenem-resistant *Enterobacterales* isolates.

CZA MIC values were substantially higher among *bla*_NDM_-positive isolates (median 256 mg/L) compared with *bla*_NDM_-negative isolates (median 2 mg/L) (Mann–Whitney U = 195, *p* < 0.001) (Figure 3). To account for the frequent co-carriage of multiple carbapenemase genes, a multivariable binary logistic regression analysis was performed using CZA resistance as the dependent variable. The overall model was statistically significant (χ^2^ = 40.4, df = 4, *p* < 0.001; McFadden’s R^2^ = 0.327). In this model, *bla*_NDM_ carriage remained independently associated with CZA resistance (adjusted OR, 52.08; 95% CI, 5.89–460.49; *p* < 0.001). In contrast, *bla*_KPC_ (adjusted OR, 0.37; 95% CI, 0.04–3.58; *p* = 0.389), *bla*_OXA-48_ (adjusted OR, 0.92; 95% CI, 0.20–4.31; *p* = 0.917), and *bla*_VIM_ (adjusted OR, 10.60; 95% CI, 0.30–372.21; *p* = 0.193) were not independently associated with CZA resistance.

Two *E. coli* isolates (2.0%), designated ES_64 and ES_73, were resistant to aztreonam–avibactam. Both AZA-resistant isolates were also resistant to ceftazidime–avibactam. ES_64 had an AZA MIC of 8 mg/L, carried *bla*_CTX-M_ and *bla*_NDM_, and showed a positive CZA–aztreonam synergy result. In contrast, ES_73 had an AZA MIC of 32 mg/L, harbored *bla*_KPC_, and did not demonstrate CZA–aztreonam synergy.

### 3.6. Whole-Genome Sequencing Findings

Whole-genome sequencing was performed for the two aztreonam–avibactam-resistant *Escherichia coli* isolates. Multilocus sequence typing identified ES_64 as ST500 and ES_73 as ST410. Assembly quality metrics were as follows: ES_64 comprised 133 contigs, with an N50 of 84,244 bp and a total assembly size of 4,790,113 bp, whereas ES_73 comprised 96 contigs, with an N50 of 221,752 bp and a total assembly size of 5,193,477 bp.

Resistome analysis revealed that ES_64 harbored *bla*_NDM-5_, *bla*_CTX-M-15_, *bla*_OXA-1_, and a chromosomal *ampC* (*BlaEC* family) β-lactamase. In contrast, ES_73 carried *bla*_KPC-3_ and *bla*_OXA-1_, together with a chromosomal *ampC* (*BlaEC* family) β-lactamase.

**Figure 4:**
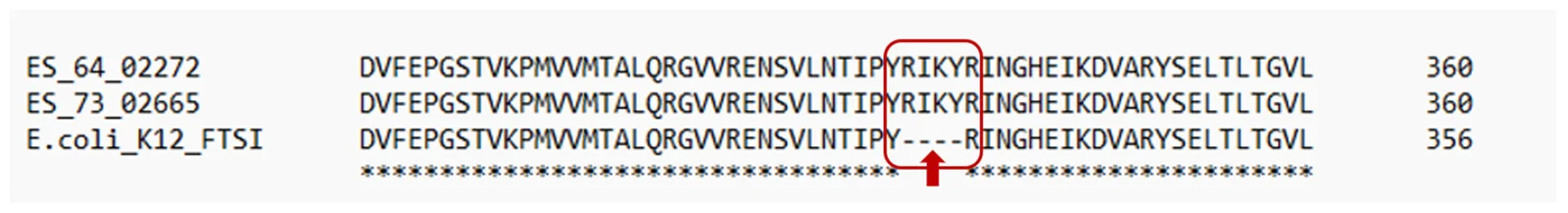
Local Clustal Omega alignment of the *FtsI*/PBP3 region surrounding the four-amino-acid insertion. Both ES_64 and ES_73 carried an identical YRIK insertion in PBP3 (after position 333) relative to the *Escherichia coli* K-12 MG1655 reference sequence. The inserted residues are boxed, and dashes indicate the corresponding gap in the reference sequence. Asterisks indicate fully conserved amino acid positions in the alignment. The complete *FtsI*/PBP3 amino acid alignment is provided as Supplementary Figure S1.

The *ftsI* (PBP3) YRIK four-amino-acid insertion was detected in both ES_64 and ES_73. Because this target-site modification was shared by both isolates, it may have contributed to reduced AZA susceptibility in both isolates but is unlikely to fully explain the higher AZA MIC observed in ES_73 compared with ES_64 (Figure 4).

Whole-genome sequencing of ES_73 followed by mapping-based variant analysis against the *Escherichia coli* K-12 MG1655 reference genome identified a 4 bp insertion within the *ompF* coding region (T→TGGAC at position NC_000913.3:986100). The insertion was supported by high sequencing depth and mapping quality (DP = 181, MQ = 60), and manual inspection of read alignments in Integrative Genomics Viewer (IGV) confirmed that the insertion was consistently present in ES_73, whereas no corresponding insertion was observed in ES_64 (Supplementary Figure S2).

**Figure 5:**
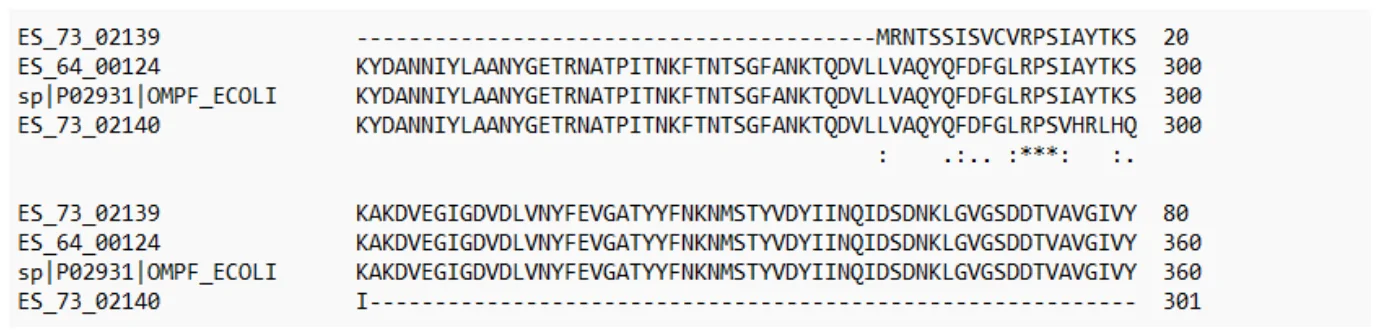
Predicted protein-level consequence of the *ompF* frameshift mutation in ES_73. Focused amino acid alignment of OmpF sequences showing disruption and premature truncation of the ES_73 OmpF sequence compared with ES_64 and the *Escherichia coli* reference OmpF protein. Asterisks indicate fully conserved amino acid positions in the alignment. Full-length alignments and IGV confirmation of the underlying 4 bp insertion are provided in Supplementary Figures S2 and S3.

Consistent with this frameshift event, de novo genome annotation of the ES_73 assembly did not identify a single intact full-length *ompF* coding sequence. Instead, the region was annotated as two overlapping partial *ompF*-like coding sequences (*ompF_1* and *ompF_2*), corresponding to a truncated OmpF-like fragment and a partial OmpF sequence. Protein-level alignment further demonstrated disruption and premature truncation of the ES_73 OmpF sequence compared with ES_64 and the reference OmpF protein (Figure 5; Supplementary Figure S3).

In contrast, no disruptive mutations were detected in the *ompF* locus of ES_64. Analysis of *ompC* in ES_73 demonstrated localized amino acid substitutions but preserved an intact open reading frame.

To explore alternative mechanisms potentially contributing to aztreonam–avibactam resistance, we screened key efflux pump regulatory genes and β-lactamase-associated loci. Variant analysis of *marR*, *acrR*, *soxR/soxS*, *ompR*, and *envZ* did not reveal disruptive mutations such as frameshift insertions or premature stop codons in either isolate. Although several single nucleotide polymorphisms were detected in ES_73, no loss-of-function alterations were identified in these regulatory genes.

Similarly, comparative analysis of the *ampC* upstream promoter/attenuator region did not demonstrate mutations suggestive of derepressed chromosomal AmpC overexpression. Both isolates carried their respective β-lactamase genes without evidence of structural disruption or promoter alterations in the chromosomal context.

These WGS findings should be interpreted as descriptive genomic observations in two AZA-resistant isolates rather than definitive evidence of causality, because AZA-susceptible comparator genomes from the same collection were not sequenced. The comparative genomic and phenotypic features of the two aztreonam–avibactam-resistant *E. coli* isolates are summarized in Table 2.

## 4. Discussion

Carbapenem-resistant *Enterobacterales* represent a major global health threat due to limited therapeutic options and the rapid emergence of complex resistance mechanisms. In this study, we evaluated the in vitro activity of ceftazidime–avibactam and aztreonam–avibactam against invasive CRE isolates collected in a tertiary-care hospital in Türkiye. The most notable finding of the present study was the markedly higher in vitro activity of aztreonam–avibactam compared with ceftazidime–avibactam, with 98% of isolates categorized as susceptible. In addition, the present study demonstrated a striking predominance of *bla*_NDM_ among the isolates, frequently detected together with *bla*_OXA-48_, reflecting the complex carbapenemase landscape that may influence the effectiveness of currently available β-lactam/β-lactamase inhibitor combinations.

In the present study, *Klebsiella pneumoniae* was the predominant species among invasive CRE isolates. This distribution was not the result of preferential selection for *K. pneumoniae*; rather, the study included consecutive, non-duplicate CRE isolates recovered from blood cultures and normally sterile body fluids during a predefined cross-sectional study period, and only the first isolate from each patient was included to avoid duplication. Therefore, the predominance of *K. pneumoniae* most likely reflects the local epidemiology of invasive CRE infections at our center, where *K. pneumoniae* is the most frequently recovered CRE pathogen. The restriction to blood cultures and sterile body fluids was intentional, as the study aimed to evaluate clinically significant invasive infections and to avoid inclusion of colonizing isolates from non-sterile specimens. However, this invasive-isolate-focused design should be considered when generalizing the species distribution to all CRE isolates encountered in routine clinical microbiology.

In the present study, aztreonam–avibactam demonstrated markedly higher in vitro activity than ceftazidime–avibactam against CRE isolates, with 98% of isolates categorized as susceptible and MIC_50/90_ values of 0.25 and 0.50 mg/L, respectively. These findings are consistent with recent European and global large-scale surveillance studies showing that aztreonam–avibactam retains potent in vitro activity against CRE isolates, including those harboring diverse carbapenemase genes, with reported MIC_50/90_ values of 0.25/0.5 mg/L and susceptibility rates exceeding 99% in European collections [14,15]. The consistently high activity of aztreonam–avibactam observed in these studies is largely attributed to the stability of aztreonam against metallo-β-lactamases combined with the inhibitory activity of avibactam against co-produced serine β-lactamases that would otherwise hydrolyze aztreonam. Together, these data support the potential role of aztreonam–avibactam as an effective therapeutic option for infections caused by carbapenem-resistant *Enterobacterales*, particularly in settings where metallo-β-lactamases such as NDM are prevalent.

Ceftazidime–avibactam was initially introduced as an effective therapeutic option against carbapenem-resistant *Enterobacterales*, particularly those producing class A and class D carbapenemases. However, its activity is limited against metallo-β-lactamase producers such as NDM, VIM, and IMP because avibactam does not inhibit class B metallo-β-lactamases [5]. This limitation has contributed to interest in combination approaches such as ceftazidime–avibactam plus aztreonam for infections caused by MBL-producing *Enterobacterales* [16]. In the present study, only 31% of isolates were susceptible to ceftazidime–avibactam, and MIC values were markedly elevated, with both MIC_50_ and MIC_90_ reaching 256 mg/L. This high resistance rate appears to be closely related to the predominance of *bla*_NDM_ detected in our isolate collection, which was present in 81%. Consistent with this observation, *bla*_NDM_ carriage showed a strong association with CZA resistance, and *bla*_NDM_-positive isolates exhibited markedly higher CZA MIC values than *bla*_NDM_-negative isolates. The reduced activity of ceftazidime–avibactam in this setting is biologically plausible, as avibactam inhibits class A, class C, and some class D β-lactamases but does not inhibit metallo-β-lactamases such as NDM. Consequently, NDM-producing *Enterobacterales* generally show resistance to ceftazidime–avibactam because avibactam does not inhibit metallo-β-lactamases. Similar findings have been reported in recent in vitro surveillance studies showing that CZA retains activity mainly against serine carbapenemase-producing isolates, particularly KPC-producing isolates, but has limited activity against MBL-producing isolates such as NDM producers [5]. Taken together, our findings highlight the substantial impact of NDM dissemination on CZA susceptibility and suggest that the spread of MBL-producing CRE may significantly compromise the clinical utility of ceftazidime–avibactam in certain geographic regions. Although the confidence intervals were wide, likely reflecting the limited number of isolates in some genotype categories, the multivariable analysis supported *bla*_NDM_ carriage as the main genomic correlate of CZA resistance in this cohort.

In Türkiye, OXA-48-like enzymes have historically been the predominant carbapenemases among CRE isolates, whereas NDM has generally been reported at substantially lower frequencies in molecular epidemiological studies from different regions of the country. However, more recent reports suggest an increasing emergence of NDM-producing isolates and strains co-harboring multiple carbapenemase genes, indicating a progressively evolving resistance landscape [17,18,19,20]. In contrast to the epidemiological pattern reported in previous studies from Türkiye, the present study demonstrated a markedly higher prevalence of *bla*_NDM_ (81%), frequently co-occurring with *bla*_OXA-48_ (72%), with dual carbapenemase production observed in 62% of isolates. These findings may suggest a possible shift toward an increased contribution of NDM-producing strains in our local epidemiology. This discrepancy may reflect local clonal expansion of NDM-producing strains or horizontal dissemination of NDM-harboring plasmids within hospital environments. This changing molecular epidemiology may have important therapeutic implications, particularly for β-lactam/β-lactamase inhibitor combinations such as ceftazidime–avibactam.

Previous non-overlapping isolate collections from our center provide useful context for interpreting the carbapenemase distribution observed in the present study. In an earlier study conducted in our center between 2015 and 2016, *bla*_OXA-48_ was the predominant carbapenemase, detected in 61% of isolates, whereas *bla*_NDM_ was rare and detected in only 3.7% of isolates, although 35% of strains harbored both enzymes [21]. In a subsequent study including isolates collected between 2017 and 2019, *bla*_OXA-48_ remained the dominant carbapenemase (87.7%), while *bla*_NDM_ increased to 23.1%, and co-production of *bla*_OXA-48_ and *bla*_NDM_ was detected in 16.9% of isolates [22]. Similarly, another study from our institution reported *bla*_OXA-48_ in 68.1% of isolates and *bla*_NDM_ in 54.3%, with co-production observed in 44% of strains [23]. In the present study, *bla*_NDM_ was detected in 81% of isolates and frequently co-occurred with *bla*_OXA-48_, with dual carbapenemase production observed in 62% of isolates. Although these findings appear to indicate a greater representation of NDM-producing and NDM/OXA-48-co-producing CRE in the current collection, direct comparisons across studies should be interpreted cautiously because of differences in study periods, isolate selection, specimen types, molecular methods, and interpretive criteria. The present study was not designed to determine the epidemiological factors underlying these differences, and the relative roles of antimicrobial selection pressure, clonal expansion, and plasmid-mediated dissemination cannot be assessed without dedicated longitudinal genomic and epidemiological analyses. Therefore, these findings should be interpreted as descriptive surveillance data supporting the need for continued molecular monitoring and antimicrobial stewardship.

The disk/gradient diffusion-based synergy assay was used as an adjunctive phenotypic assessment of the interaction between aztreonam and ceftazidime–avibactam. Zone enhancement indicating a positive interaction was observed in 98% of isolates, suggesting frequent in vitro synergy between these agents, consistent with previous data reporting synergy in 98.8% of *Klebsiella* spp. and 95% of *E. coli* isolates [13]. This interaction is biologically plausible because aztreonam is relatively stable against metallo-β-lactamase-mediated hydrolysis, whereas avibactam inhibits co-produced serine β-lactamases, including ESBLs and AmpC enzymes, that may otherwise hydrolyze aztreonam. Therefore, these findings support the microbiological rationale for aztreonam–avibactam activity in settings where NDM-producing CRE are prevalent. However, the visual CZA–aztreonam synergy result was not fully concordant with direct AZA MIC-based susceptibility categorization: one synergy-negative isolate remained AZA-susceptible with an MIC of 1 mg/L, whereas one AZA-resistant isolate with an MIC of 8 mg/L retained a detectable synergy pattern. These findings suggest that disk/gradient diffusion-based synergy testing may provide useful supportive information but should be interpreted cautiously and should not replace direct AZA MIC testing where available.

A previous study from Türkiye reported ST410 among *E. coli* isolates recovered from hospital and municipal wastewater, indicating that this lineage has previously been detected in the region [24]. However, that ecological finding is distinct from the present invasive clinical cohort and should not be interpreted as evidence of the source or route of acquisition of the clinical isolate. In the present study, ST410 was identified in an invasive carbapenem-resistant *E. coli* isolate carrying *bla*_KPC-3_, adding clinical-context data on this high-risk lineage in Türkiye. International surveillance data support the broad in vitro activity of AZA against CRE [14], whereas a recent genomic study identified AZA-resistant *E. coli* isolates, including ST410, and described a multifactorial resistance background involving PBP3 alterations and reduced outer-membrane permeability [25].

Whole-genome sequencing of the aztreonam–avibactam-resistant isolates identified several genomic features previously linked to reduced aztreonam or aztreonam–avibactam susceptibility. Both isolates carried a four-amino-acid insertion (YRIK) in the penicillin-binding protein 3 encoded by the *ftsI* gene. Insertions such as YRIN or YRIK in PBP3 have previously been associated with reduced susceptibility to aztreonam and aztreonam–avibactam in *Enterobacterales*, as they alter the target site of β-lactam antibiotics and decrease binding affinity [26]. Previous studies have demonstrated that decreased susceptibility to aztreonam–avibactam may result from alterations in penicillin-binding protein 3 together with reduced outer membrane permeability and the presence of additional β-lactamases. Recent genomic analyses of clinical *Escherichia coli* isolates have shown that PBP3 insertions and impermeability mechanisms can contribute to elevated aztreonam–avibactam MIC values, particularly when combined with other resistance determinants [25]. Accordingly, we additionally investigated genes involved in porin expression, efflux regulation, and chromosomal AmpC regulation. Although no disruptive mutations were identified in these loci, this analysis did not provide genomic evidence for several previously proposed mechanisms associated with reduced aztreonam–avibactam susceptibility.

This study has several strengths. It provides clinically relevant data based on consecutive, non-duplicate invasive CRE isolates collected in a tertiary-care center and directly compares ceftazidime–avibactam and aztreonam–avibactam within the same isolate collection. In addition, the integration of phenotypic susceptibility testing, carbapenemase gene profiling, and multivariable genotype–phenotype analysis allowed assessment of the relationship between carbapenemase carriage and CZA resistance in a setting with frequent carbapenemase co-carriage. Another strength is the parallel evaluation of direct AZA MIC testing and CZA–aztreonam synergy testing, which provided practical information on the interpretation of qualitative synergy assays. Whole-genome sequencing of the two AZA-resistant *E. coli* isolates enabled descriptive characterization of genomic features previously associated with reduced aztreonam–avibactam susceptibility. Finally, because aztreonam–avibactam was not routinely available in Türkiye during the study period, this study provides local baseline data from invasive CRE isolates before potential routine clinical implementation. To the best of our knowledge, no previous study from Türkiye has combined phenotypic AZA/CZA susceptibility testing, carbapenemase gene profiling, synergy testing, and WGS-based characterization in a single-center cohort of invasive CRE isolates.

The main limitations of this study should be acknowledged. This was a single-center study of invasive CRE isolates, and the predominance of *Klebsiella pneumoniae* limited species-specific comparisons. Aztreonam–avibactam MICs were determined by gradient diffusion and were not confirmed by broth microdilution; therefore, MIC values close to the EUCAST susceptibility breakpoint should be interpreted cautiously, particularly in isolates carrying PBP3 insertions. Clinical outcome data, clonal relatedness analysis of the full isolate collection, and plasmid characterization were not available, limiting assessment of clinical correlates and dissemination mechanisms underlying the observed resistance patterns, including the high rate of *bla*_NDM_/*bla*_OXA-48_ co-carriage. Finally, whole-genome sequencing was limited to the two AZA-resistant *E. coli* isolates and was not complemented by AZA-susceptible comparator genomes, gene expression analysis, or functional validation experiments; therefore, the WGS findings should be interpreted as descriptive genomic observations rather than definitive mechanistic evidence.

## 5. Conclusions

In conclusion, because aztreonam–avibactam was not listed among licensed human medicinal products in Türkiye during the study period, this study provides clinically relevant local baseline susceptibility data on AZA MIC distributions among invasive CRE isolates before routine clinical implementation of AZA in the country. Our findings demonstrate that AZA retains potent in vitro activity against invasive CRE isolates in our setting, including a collection characterized by a high prevalence of *bla*_NDM_ and frequent carbapenemase co-production. The observed discordance between qualitative CZA–aztreonam synergy testing and direct AZA MIC-based susceptibility categorization further indicates that synergy assays should be interpreted cautiously and should not replace direct AZA MIC determination where available. In contrast, the predominance of *bla*_NDM_ substantially limits the utility of ceftazidime–avibactam, highlighting the clinical relevance of local carbapenemase epidemiology when selecting treatment strategies. The observed shift toward increasing NDM prevalence further emphasizes the need for continuous molecular surveillance to monitor evolving resistance patterns and to guide antimicrobial stewardship in regions where metallo-β-lactamase-producing CRE are emerging.

## Figures and Tables

**Table 1:** Ceftazidime–Avibactam Susceptibility According to Carbapenemase Gene Category.

Carbapenemase Gene Category	Gene-Positive İsolates, *N*	CZA-Resistant İsolates, *n*/*N* (%)
*bla*_NDM_-positive	81	67/81 (82.7%)
*bla*_OXA-48_-positive	72	54/72 (75.0%)
*bla*_KPC_-positive	14	3/14 (21.4%)
*bla*_VIM_-positive	4	3/4 (75.0%)
No carbapenemase detected	1	0/1 (0%)

Carbapenemase gene categories are not mutually exclusive. Isolates co-harboring multiple carbapenemase genes were included in each relevant gene-positive category; therefore, row counts should not be summed.

**Table 2:** Comparative Genomic and Phenotypic Features of the Two Aztreonam–Avibactam-Resistant *E. coli* Isolates.

Category	Feature	ES_64	ES_73
**Phenotype**	Aztreonam–avibactam MIC (mg/L)	8	32
	Ceftazidime–avibactam MIC (mg/L)	12	256
**Clonal background**	MLST (Achtman scheme)	ST500	ST410
**Carbapenemase**		*blaNDM-5*	*blaKPC-3*
**Other acquired β-lactamases**		*blaCTX-M-15*, *blaOXA-1*	*blaOXA-1*
**Chromosomal β-lactamase**		*blaEC* (*ampC*)	*blaEC* (*ampC*)
**Target modification**	PBP3 (ftsI)	YRIK insertion	YRIK insertion
**Outer membrane permeability**	OmpF	Intact	4 bp insertion
	OmpC	Intact ORF	Amino acid substitutions (intact ORF)
**Efflux regulation**	*marR*, *acrR*, *soxR/soxS*, *ompR/envZ*	No disruptive mutations	No disruptive mutations

The synergy-negative isolate with an AZA MIC of 1 mg/L was AZA-susceptible and was not included in this WGS table.

## Data Availability

The normalized VCF files and gene-level variant tables generated during whole-genome sequencing analysis are provided as Supplementary Data S1 (Supplementary_Data.zip). All analyses were performed against the *Escherichia coli* K-12 MG1655 reference genome (NC_000913.3). Raw sequencing reads are available from the corresponding author upon reasonable request.

## Supplementary Materials

The following supporting information can be downloaded at: https://www.mdpi.com/article/10.3390/antibiotics15080724/s1, Figure S1: Full-length Clustal Omega multiple sequence alignment of FtsI/PBP3 amino acid sequences from ES_64, ES_73, and *E. coli* K-12 MG1655; Figure S2: IGV visualization of the ompF insertion in ES_73; Figure S3: Full Clustal Omega alignment of OmpF amino acid sequences.
