# Supplementary material for: Combating Carbapenem-Resistant Enterobacterales: Comparative In Vitro Efficacy of Aztreonam–Avibactam and Ceftazidime–Avibactam and Distribution of Carbapenemase Genes
Source: Antibiotics (Basel). 2026 Jul 25;15(8):724. doi: 10.3390/antibiotics15080724 (PMC13509184; doi:10.3390/antibiotics15080724)
Supplement: Supplementary file 1 [file antibiotics-15-00724-s001.zip › Supplementary Figure S1.pdf]

```
CLUSTAL O(1.2.4) multiple sequence alignment

ES_64_02272         MKAAAKTQKPKRQEEHANFISWRFALLCGCILLALAFLLGRVAWLQVISPDMLVKEGDMR      60
ES_73_02665         MKAAAKTQKPKRQEEHANFISWRFALLCGCILLALAFLLGRVAWLQVISPDMLVKEGDMR      60
E.coli_K12_FTSI     MKAAAKTQKPKRQEEHANFISWRFALLCGCILLALAFLLGRVAWLQVISPDMLVKEGDMR      60
                    ************************************************************

ES_64_02272         SLRVQQVSTSRGMITDRSGRPLAVSVPVKAIWADPKEVHDAGGISVGDRWKALANALNIP      120
ES_73_02665         SLRVQQVSTSRGMITDRSGRPLAVSVPVKAIWADPKEVHDAGGISVGDRWKALANALNIP      120
E.coli_K12_FTSI     SLRVQQVSTSRGMITDRSGRPLAVSVPVKAIWADPKEVHDAGGISVGDRWKALANALNIP      120
                    ************************************************************

ES_64_02272         LDQLSARINANPKGRFIYLARQVNPDMADYIKKLKLPGIHLREESRRYYPSGEVTAHLIG      180
ES_73_02665         LDQLSARINANPKGRFIYLARQVNPDMADYIKKLKLPGIHLREESRRYYPSGEVTAHLIG      180
E.coli_K12_FTSI     LDQLSARINANPKGRFIYLARQVNPDMADYIKKLKLPGIHLREESRRYYPSGEVTAHLIG      180
                    ************************************************************

ES_64_02272         FTNVDSQGIEGVEKSFDKWLTGQPGERIVRKDRYGRVIEDISSTDSQAAHNLALSIDERL      240
ES_73_02665         FTNVDSQGIEGVEKSFDKWLTGQPGERIVRKDRYGRVIEDISSTDSQAAHNLALSIDERL      240
E.coli_K12_FTSI     FTNVDSQGIEGVEKSFDKWLTGQPGERIVRKDRYGRVIEDISSTDSQAAHNLALSIDERL      240
                    ************************************************************

ES_64_02272         QALVYRELNNAVAFNKAESGSAVLVDVNTGEVLAMANSPSYNPNNLSGTPKEAMRNRTIT      300
ES_73_02665         QALVYRELNNAVAFNKAESGSAVLVDVNTGEVLAMANSPSYNPNNLSGTPKEAMRNRTIT      300
E.coli_K12_FTSI     QALVYRELNNAVAFNKAESGSAVLVDVNTGEVLAMANSPSYNPNNLSGTPKEAMRNRTIT      300
                    ************************************************************

ES_64_02272         DVFEPGSTVKPMVVMTALQRGVVRENSVLNTIPYRIKYRINGHEIKDVARYSELTLTGVL      360
ES_73_02665         DVFEPGSTVKPMVVMTALQRGVVRENSVLNTIPYRIKYRINGHEIKDVARYSELTLTGVL      360
E.coli_K12_FTSI     DVFEPGSTVKPMVVMTALQRGVVRENSVLNTIPY----RINGHEIKDVARYSELTLTGVL      356
                    **********************************    **********************

ES_64_02272         QKSSNVGVSKLALAMPSSALVDTYSRFGLGKATNLGLVGERSGLYPQKQRWSDIERVTFS      420
ES_73_02665         QKSSNVGVSKLALAMPSSALVDTYSRFGLGKATNLGLVGERSGLYPQKQRWSDIERVTFS      420
E.coli_K12_FTSI     QKSSNVGVSKLALAMPSSALVDTYSRFGLGKATNLGLVGERSGLYPQKQRWSDIERATFS      416
                    ********************************************************.***

ES_64_02272         FGYGLMVTPLQLARVYATIGSYGIYRPLSITKVDPPVPGERVFPESIVRTVVHMMESVAL      480
ES_73_02665         FGYGLMVTPLQLARVYATIGSYGIYRPLSITKVDPPVPGERVFPESIVRTVVHMMESVAL      480
E.coli_K12_FTSI     FGYGLMVTPLQLARVYATIGSYGIYRPLSITKVDPPVPGERVFPESIVRTVVHMMESVAL      476
                    ************************************************************

ES_64_02272         PGGGGVKAAIKGYRIAIKTGTAKKVGPDGRYINKYIAYTAGVAPASQPRFALVVVINDPQ      540
ES_73_02665         PGGGGVKAAIKGYRIAIKTGTAKKVGPDGRYINKYIAYTAGVAPASQPRFALVVVINDPQ      540
E.coli_K12_FTSI     PGGGGVKAAIKGYRIAIKTGTAKKVGPDGRYINKYIAYTAGVAPASQPRFALVVVINDPQ      536
                    ************************************************************

ES_64_02272         AGKYYGGAVSAPVFGAIMGGVLRTMNIEPDALTTGDKNEFVINQGEGTGGRS      592
ES_73_02665         AGKYYGGAVSAPVFGAIMGGVLRTMNIEPDALTTGDKNEFVINQGEGTGGRS      592
E.coli_K12_FTSI     AGKYYGGAVSAPVFGAIMGGVLRTMNIEPDALTTGDKNEFVINQGEGTGGRS      588
                    ****************************************************
```

Supplementary Figure S1. Full-length Clustal Omega multiple sequence alignment of FtsI/PBP3 amino acid sequences from ES_64, ES_73, and *E. coli* K-12 MG1655.
