# Supplementary material for: Combating Carbapenem-Resistant Enterobacterales: Comparative In Vitro Efficacy of Aztreonam–Avibactam and Ceftazidime–Avibactam and Distribution of Carbapenemase Genes
Source: Antibiotics (Basel). 2026 Jul 25;15(8):724. doi: 10.3390/antibiotics15080724 (PMC13509184; doi:10.3390/antibiotics15080724)
Supplement: Supplementary file 1 [file antibiotics-15-00724-s001.zip › Supplementary Figure S2.pdf]

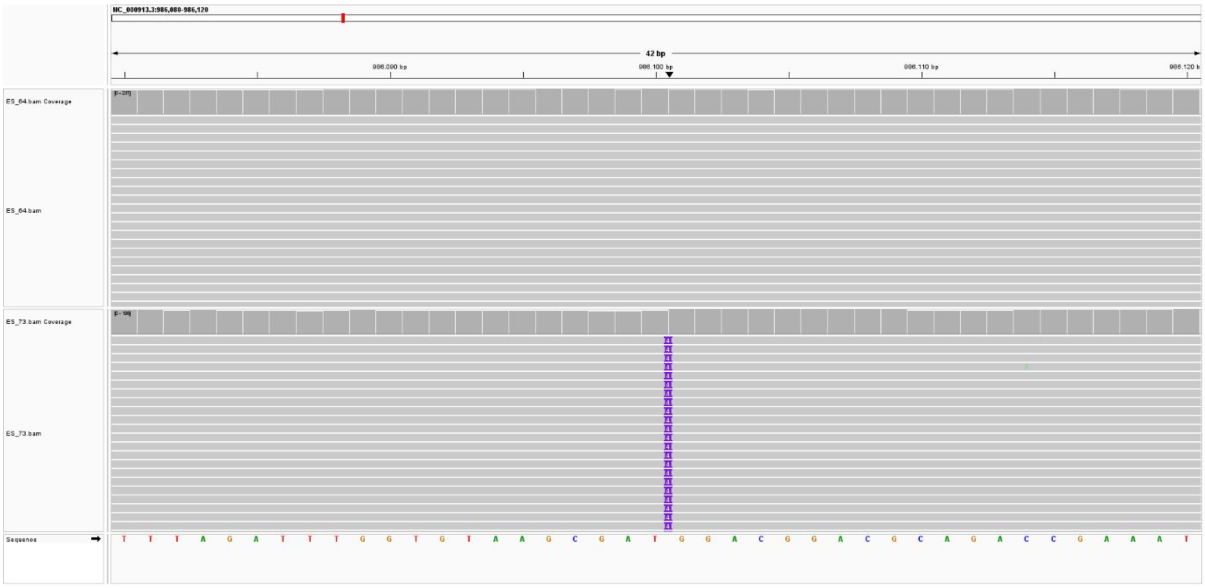

Supplementary Figure S2. IGV visualization of the ompF **insertion in ES_73.** Integrative Genomics Viewer snapshot showing read alignments of ES_64 and ES_73 mapped to the *Escherichia coli* K-12 MG1655 reference genome at the *ompF* locus. ES_73 harbored a 4-bp insertion at position NC_000913.3:986100, whereas ES_64 showed no insertion at the corresponding site. The variant was supported by high sequencing depth and mapping quality (DP = 181, MQ = 60).
