# Supplementary material for: Combating Carbapenem-Resistant Enterobacterales: Comparative In Vitro Efficacy of Aztreonam–Avibactam and Ceftazidime–Avibactam and Distribution of Carbapenemase Genes
Source: Antibiotics (Basel). 2026 Jul 25;15(8):724. doi: 10.3390/antibiotics15080724 (PMC13509184; doi:10.3390/antibiotics15080724)
Supplement: Supplementary file 1 [file antibiotics-15-00724-s001.zip › Supplementary Figure S3.pdf]

```
CLUSTAL O(1.2.4) multiple sequence alignment


ES_73_02139               ------------------------------------------------------------   0
ES_64_00124               MMKRNILAVIVPALLVAGTANAAEIYNKDGNKVDLYGKAVGLHYFSKGNGENSYGGNGDM   60
sp|P02931|OMPF_ECOLI      MMKRNILAVIVPALLVAGTANAAEIYNKDGNKVDLYGKAVGLHYFSKGNGENSYGGNGDM   60
ES_73_02140               MMKRNILAVIVPALLVAGTANAAEIYNKDGNKVDLYGKAVGLHYFSKGNGENSYGGNGDM   60


ES_73_02139               ------------------------------------------------------------   0
ES_64_00124               TYARLGFKGETQINSDLTGYGQWEYNFQGNNSEGADAQTGNKTRLAFAGLKYADVGSFDY   120
sp|P02931|OMPF_ECOLI      TYARLGFKGETQINSDLTGYGQWEYNFQGNNSEGADAQTGNKTRLAFAGLKYADVGSFDY   120
ES_73_02140               TYARLGFKGETQINSDLTGYGQWEYNFQGNNSEGADAQTGNKTRLAFAGLKYADVGSFDY   120


ES_73_02139               ------------------------------------------------------------   0
ES_64_00124               GRNYGVVYDALGYTDMLPEFGGDTAYSDDFFVGRVGGVATYRNSNFFGLVDGLNFAVQYL   180
sp|P02931|OMPF_ECOLI      GRNYGVVYDALGYTDMLPEFGGDTAYSDDFFVGRVGGVATYRNSNFFGLVDGLNFAVQYL   180
ES_73_02140               GRNYGVVYDALGYTDMLPEFGGDTAYSDDFFVGRVGGVATYRNSNFFGLVDGLNFAVQYL   180


ES_73_02139               ------------------------------------------------------------   0
ES_64_00124               GKNERDTARRSNGDGVGGSISYEYEGFGIVGAYGAADRTNLQEAQPLGNGKKAEQWATGL   240
sp|P02931|OMPF_ECOLI      GKNERDTARRSNGDGVGGSISYEYEGFGIVGAYGAADRTNLQEAQPLGNGKKAEQWATGL   240
ES_73_02140               GKNERDTARRSNGDGVGGSISYEYEGFGIVGAYGAADRTNLQEAQPLGNGKKAEQWATGL   240


ES_73_02139               ----------------------------------------MRNTSSISVCVRPSIAYTKS   20
ES_64_00124               KYDANNIYLAANYGETRNATPITNKFTNTSGFANKTQDVLLVAQYQFDFGLRPSIAYTKS   300
sp|P02931|OMPF_ECOLI      KYDANNIYLAANYGETRNATPITNKFTNTSGFANKTQDVLLVAQYQFDFGLRPSIAYTKS   300
ES_73_02140               KYDANNIYLAANYGETRNATPITNKFTNTSGFANKTQDVLLVAQYQFDFGLRPSVHRLHQ   300
                                                                  :    .:.. :***:   :.

ES_73_02139               KAKDVEGIGDVDLVNYFEVGATYYFNKNMSTYVDYIINQIDSDNKLGVGSDDTVAVGIVY   80
ES_64_00124               KAKDVEGIGDVDLVNYFEVGATYYFNKNMSTYVDYIINQIDSDNKLGVGSDDTVAVGIVY   360
sp|P02931|OMPF_ECOLI      KAKDVEGIGDVDLVNYFEVGATYYFNKNMSTYVDYIINQIDSDNKLGVGSDDTVAVGIVY   360
ES_73_02140               I-----------------------------------------------------------   301


ES_73_02139               QF    82
ES_64_00124               QF    362
sp|P02931|OMPF_ECOLI      QF    362
ES_73_02140               --    301
```

Supplementary Figure S3. Full Clustal Omega alignment of OmpF amino acid sequences.
